# Supplementary material for: Splice-Junction-Based Mapping of Alternative Isoforms in the Human Proteome
Source: Cell Rep. Author manuscript; Available in PMC 2020 Jan 15. (PMC6961840; doi:10.1016/j.celrep.2019.11.026)
Supplement: 3 [file NIHMS1546469-supplement-3.zip › DF2/PXD009737/TestisTrypsin-31-Q13428-KQEGPATQVDSAVGTLPATSPQSTSVQAK.pdf]

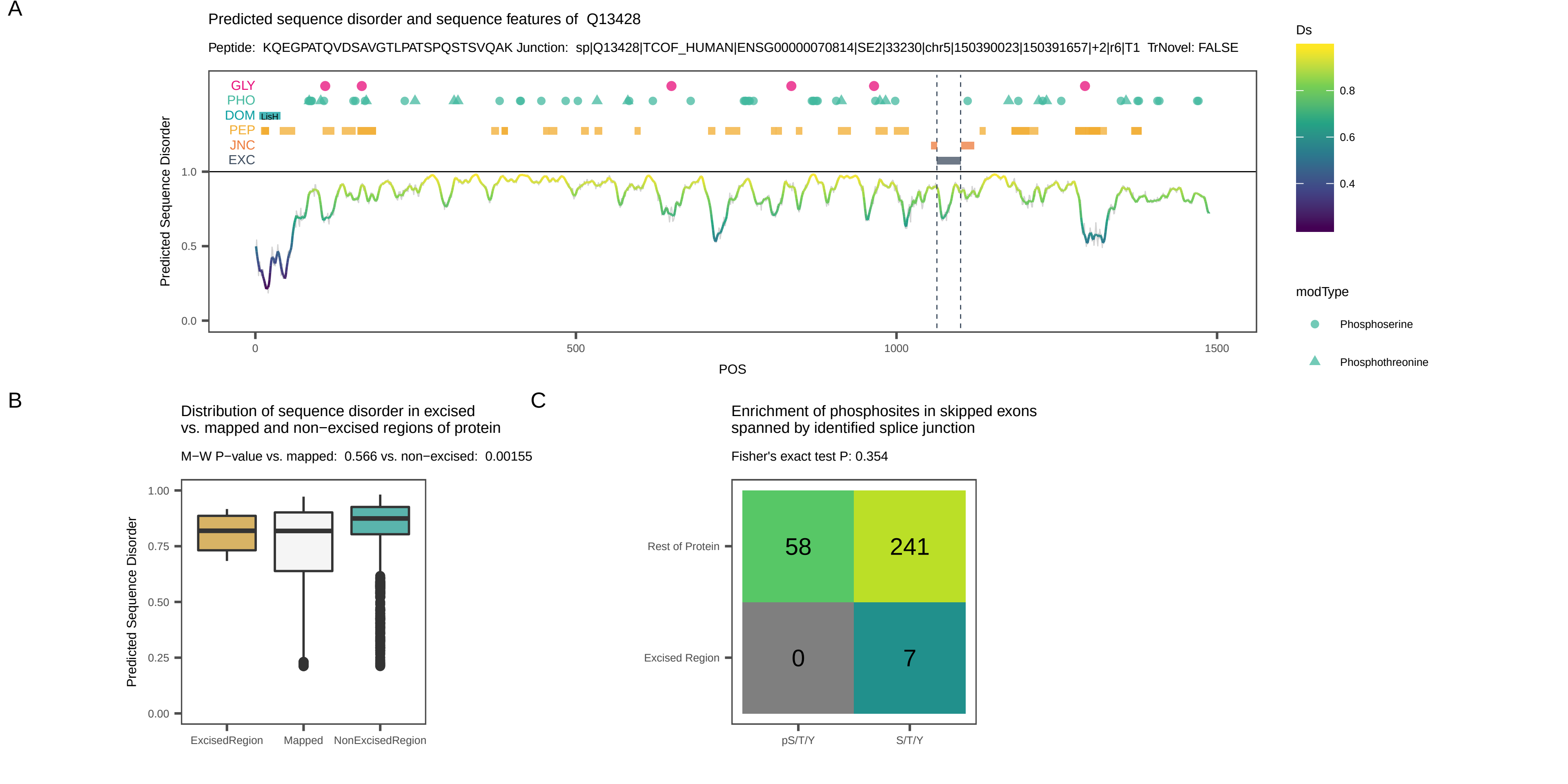

A
Predicted sequence disorder and sequence features of Q13428
Peptide: KQEGPATQVDSAVGTLPATSPQSTSVQAK Junction: sp|Q13428|TCOF_HUMAN|ENSG00000070814|SE2|33230|chr5|150390023|150391657|+2|r6|T1 TrNovel: FALSE
GLY
PHO
DOM
LisH
PEP
JNC
EXC
Predicted Sequence Disorder
1.0
0.5
0.0
0
500
1000
1500
POS
Ds
0.8
0.6
0.4
modType
Phosphoserine
Phosphothreonine
B
Distribution of sequence disorder in excised vs. mapped and non−excised regions of protein
M−W P−value vs. mapped: 0.566 vs. non−excised: 0.00155
Predicted Sequence Disorder
1.00
0.75
0.50
0.25
0.00
ExcisedRegion
Mapped
NonExcisedRegion
C
Enrichment of phosphosites in skipped exons spanned by identified splice junction
Fisher's exact test P: 0.354
Rest of Protein
Excised Region
58
241
0
7
pS/T/Y
S/T/Y
